# Experimental transmission of *Babesia microti* by *Rhipicephalus haemaphysaloides*

**DOI:** 10.1186/s13071-016-1517-2

**Published:** 2016-04-25

**Authors:** Lan-Hua Li, Dan Zhu, Chen-Chen Zhang, Yi Zhang, Xiao-Nong Zhou

**Affiliations:** Key Laboratory of Parasite & Vector Biology, Ministry of Health, National Institute of Parasitic Diseases, Chinese Center for Disease Control and Prevention, WHO Collaborating Centre for Malaria, Schistosomiasis and Filariasis, Shanghai, 200025 People’s Republic of China; School of Publish Health and Management, Weifang Medical University, Weifang, 261053 PR China

**Keywords:** *Babesia microti*, *Rhipicephalus haemaphysaloides*, Transmission, Vector competence

## Abstract

**Background:**

Human babesiosis is considered an emerging threat in China. Dozens of human infections with *Babesia microti* have been reported recently, especially in southern China. However, the transmission vectors of this parasite in these areas are not well understood. *Rhipicephalus haemaphysaloides,* which is one of the dominant tick species in southern China, is a major vector of bovine babesiosis in China. However, whether this tick has the potential to transmit *B. microti* has not been tested. The present study experimentally investigated the transmission competence of *B. microti* through *R. haemaphysaloides* ticks.

**Methods:**

Larvae and nymphs of *R. haemaphysaloides* ticks were fed on laboratory mice infected by *B. microti*. The infection was detected by PCR at 4 weeks post-molting. BALB/c and NOD/SCID mice were infested by nymphs molting from larvae that ingested the blood of infective mice, and blood samples were then analyzed by PCR.

**Results:**

Experimental transstadial transmission of *R. haemaphysaloides* for *B. microti* was proved in both the larvae to nymph and the nymph to adult transstadial routes. The positive rate of *B. microti* was 43.8 % in nymphs developed from larvae consumed infected mice and 96.7 % in adults developed from nymphs exposed to positive mice. Among the mice infested by infective nymphs, *B. microti* was detected in 16.7 % (2/12) of the BALB/c mice and in all of the NOD/SCID (6/6). However, the parasite was not observed to persist beyond more than one molt, and transovarial transmission did not occur.

**Conclusions:**

This is the first study to demonstrate that *B. microti* can be transmitted artificially by *R. haemaphysaloides*. This tick species might be a potential vector of human babesiosis in southern China, which represents a public health concern.

## Background

*Babesia microti* is a tick-transmitted, intraerythrocytic parasite that usually infects wild animals worldwide. This parasite is also known to be one of the major causative agents of human babesiosis and endemic mainly in the northeastern and upper midwestern United States [1]. Sporadic cases of *B. microti* or *B. microti*-like organisms have also been documented in places such as Germany, Japan, South Korea and Mongolia [1, 2]. In China, human cases of *B. microti* have been increasingly reported in recent years. In 2013, Zhou et al. reported 11 cases in Yunnan Province of southwestern China [3–5]. In 2015, Qiao et al. diagnosed a babesiosis patient and found 40 infected cases in colleagues of the patient in Guangxi Province of southern China [6]. Moreover, most medical staff are unaware of the risk, and investigation of this risk in the mass population is scarce. Hence, the number of human infections with this parasite might be largely underestimated. Together with the fact that human infections by other *babesia* species have also increased significantly in China [7, 8], it is believed that babesiosis is an emerging threat in the country [9].

Ticks of the genus *Ixodes* are considered the primary vector of *B. microti* [10]. The parasite is mainly transmitted to people by *Ixodes scapularis* in the United States. *Ixodes spinipalpis* is also a known vector, and still other *Ixodes* spp., including *I. angustus*, *I. eastoni* and *I. muris*, are suspected to transmit the parasite in North America. In many European countries, the primary vector of *B. microti* is usually *I. ricinus*, although *I. trianguliceps* is believed to play a more important role in England [11]. Infections by *I. persulcatus* have also been reported in Russia. In Asia, *B. microti* Hobetsu type have been detected in questing *I. ovatus* from Japan, and US type and a type related to *B. microti* Kobe have been detected in *I. persulcatus* [10, 12–15]. There are few studies on the vectors of *B. microti* in China, except in the northeastern regions, where *I. persulcatus* is considered the primary vector [13, 16]. However, competent vectors of *B. microti* in other areas of the country are not yet well understood [17].

Interestingly, *B. microti* was recently detected in ticks outside the *Ixodes* genus. Wójcik-Fatla and colleagues detected infections in questing *Dermacentor reticulatus* ticks from Poland [18], and the parasite was reported in questing *Haemaphysalis concinna* ticks from northeast China [16]. Therefore, more studies are needed on the transmission potentials of tick species from other genera.

*Rhipicephalus haemaphysaloides*, a three-host hard tick, is widely distributed in southern China and other countries in Southeast Asia [17, 19]. This species has been reported to be a major vector of bovine babesiosis in China [20] and can transmit the Kyasanur Forest disease virus [21]. As far as we are aware, the transmission of *B. microti* through *R. haemaphysaloides* has not been reported. To understand whether *R. haemaphysaloides* could play a role in the spread of *B. microti* in China, we performed a series of experiments to study the transmission competence of *B. microti* through a *R. haemaphysaloides* strain maintained in our laboratory.

## Methods

### Experimental mice and ticks

Specific-pathogen-free (SPF) female BALB/c or NOD/SCID mice weighing 16 to 18 grams were purchased from Shanghai Laboratory Animal Center, Chinese Academy of Sciences. All animal experiments were performed according to the protocols approved by the Ethics Committee at the National Institute of Parasitic Diseases, Chinese Center for Disease Control and Prevention based in Shanghai.

An engorged *Rhipicephalus haemaphysaloides* female was removed from a dog in a village of Tengchong County, Yunnan province, China, in 2014. The tick was then maintained in our laboratory in an incubator at 25 °C, with 85 % relative humidity and a 14/10 h light/dark photoperiod regimen. The larvae and nymphs of *R. haemaphysaloides* used in this study were the third generation of the colony.

### Transmission experiments of *Babesia microti*

**Fig. 1:**
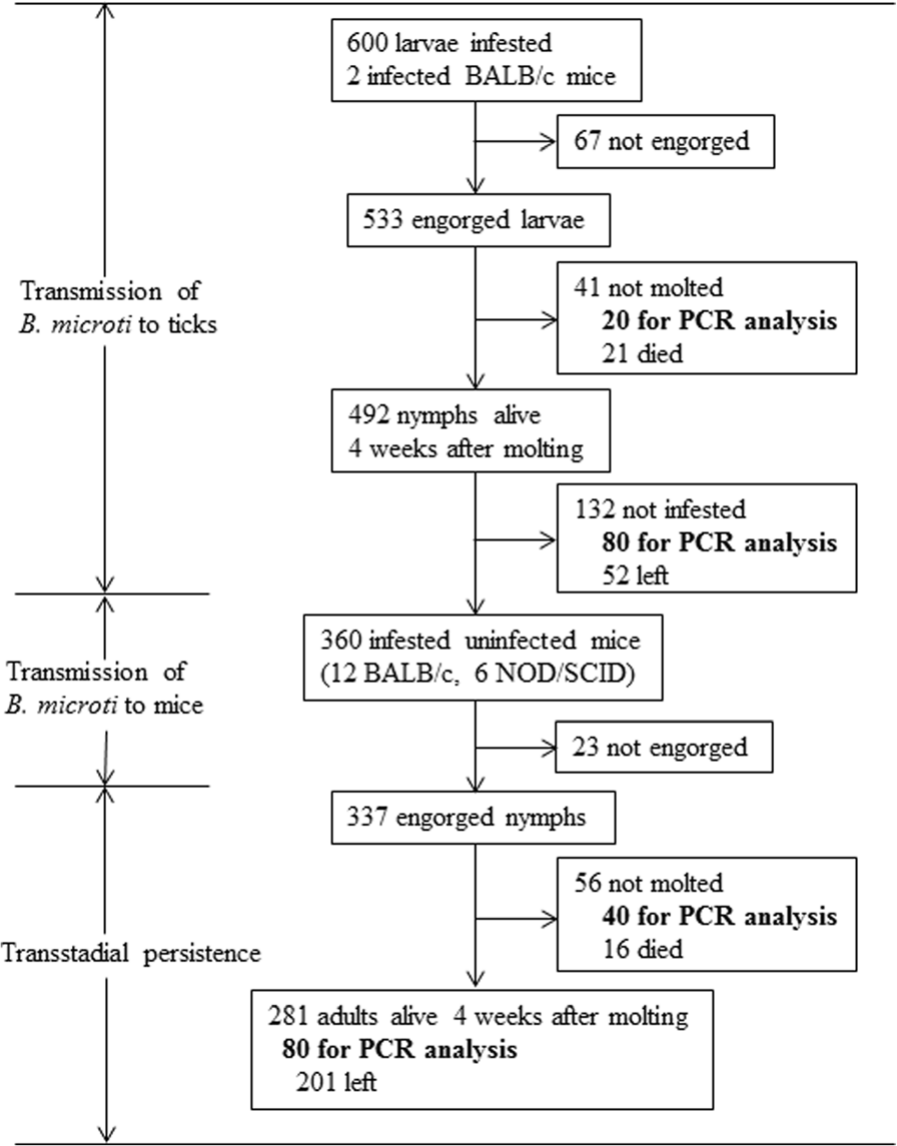
The investigation diagram with the results for the transmission experiments of *B. microti* through *R. haemaphysaloides* larvae

**Fig. 2:**
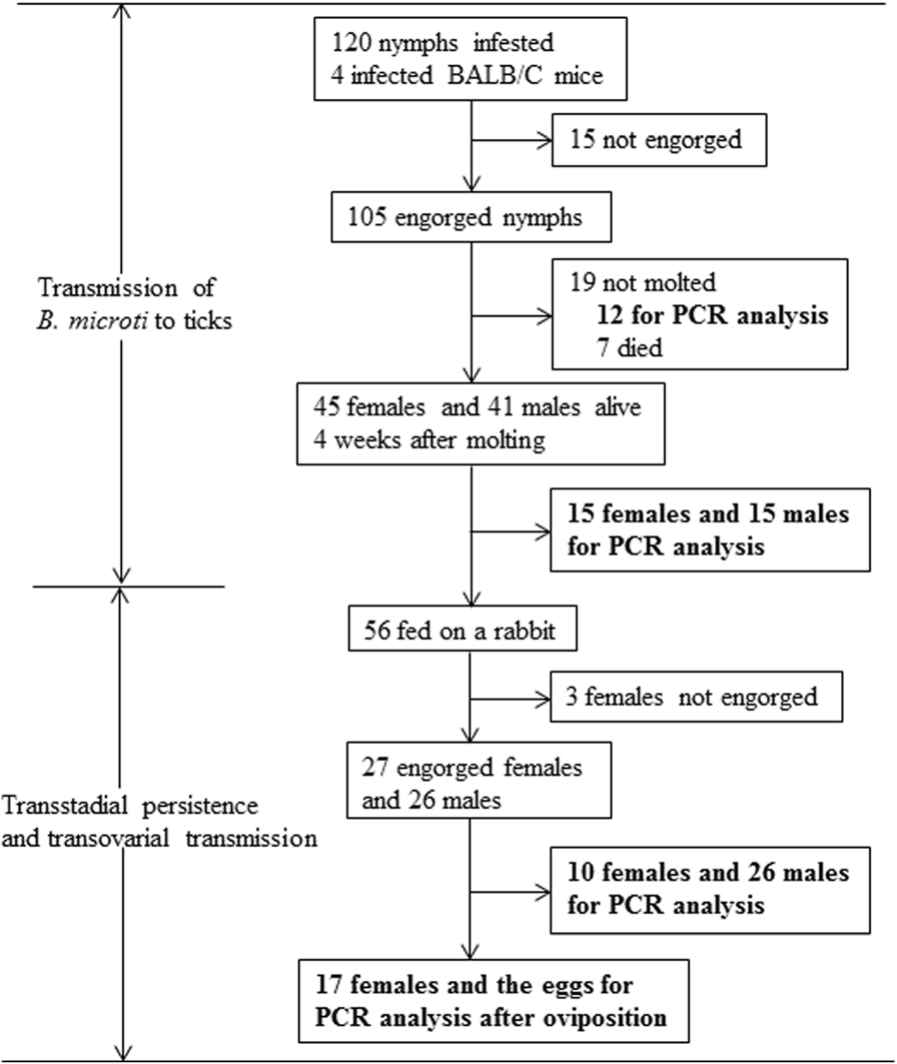
The investigation diagram with the results for transmission experiments of *B. microti* through *R. haemaphysaloides* nymphs

Three experiments were performed to study the transmission competence of *R. haemaphysaloides* ticks. The experimental procedures are shown in Figs. 1 and 2.

#### **(i)Transmission of*B. microti*from mice to ticks**

*B. microti* (Peabody mjr Strain, ATCC PRA-99) [22] were provided by Institute of Laboratory Animal Sciences, Chinese Academy of Sciences. The cryopreserved stabilates of *B. microti* were thawed to room temperature. Donor BALB/c mice were infected intraperitoneally with 100 μl of the stabilates. The resulting infections were then inoculated intraperitoneally into experimental animals at doses of 100 μl of blood with 50–60 % parasitemia.

Approximately 600 larvae were applied equally by brush to 2 mice with *B. microti* parasitemia of 20–30 % three days after incubation. Each mouse was collared with a plastic cap with central drilling to prevent grooming. In the same way, 4 infected mice were infested with nymphs by applying 30 nymphs to each animal. Mice infested with ticks were maintained over trays with water in the bottom from which detached engorged ticks were harvested.

Twenty engorged larvae (10 from each mouse) and 12 engorged nymphs (3 from each mouse) were placed in 70 % alcohol for storage before being analyzed for infection by PCR. The engorged ticks remaining were maintained in 6 bottles at 25 °C, with 85 % relative humidity and a 14/10 h light/dark photoperiod regimen.

Twenty-eight days after the first molted tick was observed, 80 nymphs or 30 adults (15 females and 15 males) were analyzed for infection by PCR. The remaining ticks were used for the following experiments.

#### **(ii)Transmission of*B. microti*to mice by infected nymphs**

Twelve BALB/c mice or 6 NOD/SCID mice were each infested with 20 nymphs that were infected with *B. microti* as larvae. Approximately 50 μl tail blood of mice was collected for *B. microti* detection on every other day from day 7 to 30 after infestation. For each blood sample, a thin blood smear was made with 2 μl of blood, fixed with methanol, Giemsa-stained and examined using a microscope. Forty-five microliters of blood was used for DNA extraction, and PCR was performed to examine *B. microti* infection. Blood collection was stopped after the mice were proved to be infected by PCR. The engorged nymphs were used for the transstadial persistence experiment.

#### **(iii)Transstadial persistence and transovarial transmission experiments**

Forty engorged nymphs dropped from the uninfected BALB/c mice in the second experiment were stored for PCR analysis. The remaining ticks were maintained for molting as described above. Twenty-eight days after the first molted tick was observed, 80 adults harvested were analyzed by PCR to investigate transstadial persistence.

The infected adult ticks obtained in the first experiment were fed on the shaved ears of a laboratory rabbit. Ten engorged females and all of the fed males were stored for PCR. The other engorged females were maintained in bottles for oviposition and then stored for PCR analysis. Transovarial transmission was investigated by examining the resulting eggs and larvae by PCR.

### DNA extraction

DNA was extracted from both tick samples and mouse blood using a DNeasy Blood & Tissue Kit (Qiagen, Hilden, Germany) according to the manufacturer’s protocol. DNA was extracted in pools of approximately 50 eggs from both the infected females and the resulting larvae and was extracted individually for other ticks.

### PCR assay

Nested PCR was used to test for infection using a set of primers highly specific for *B. microti*. Partial fragments of 18S rRNA gene were amplified using the primer sets Bab1A (5′- GTCTTAGTATAAGCTTTTATACAGCG-3′) and Bab4A (5′-GATAGGTCAGAAACTTGAATGATACATCG-3′) for the first round and Bab2A (5′-CAGTTATAGTTTATTTGATGTTCGTTTTAC-3′) and Bab3A (5′-CGGCAAAGCCATGCGATTCGCTAAT-3′) for the second round, as described by Zamoto et al. [23]. Negative and positive controls were always used in each PCR reaction. One negative control was used in each row of a 96-well PCR plate or 8-tube strip to exclude contamination. PCR was performed in a C1000 Touch™ Thermal Cycler (Bio-Rad laboratories Incorporation, California, USA) starting with a pre-PCR heat step of 3 min at 95 °C, followed by 35 cycles of 94 °C for 40 s, 57 and 72 °C for 30 s, and ending at 72 °C for 5 min. Samples with 154-bp PCR products were recognized as *B. microti* - positive.

## Results

### Transmission of *B. microti* to ticks by infected mice

Among the 600 larvae of *R. haemaphysaloides* infesting mice that were infected with *B. microti*, 533 were engorged and collected (Fig. 1). Nineteen of the 20 (95.5 %) engorged larvae were positive by PCR, which demonstrates that the ticks had ingested the parasite (Table 1). Eighty of the 492 molted nymphs were analyzed for infection 4 weeks after molting, and 35 (43.8 %) were positive.

**Table 1:** Infection of *R. haemaphysaloides* by exposure to mice infected with *B. microti*

Tick stage applied	No. of engorged ticks analyzed	No. of positive engorged ticks (%)	No. of ticks analyzed 4 week post molting	No. of positive molted ticks (%)*
Larva	20	19 (95.5)	80	35 (43.8)
Nymph	12	12 (100)	30	29 (96.7)

*P < 0.01

A total of 105 engorged nymphs were collected from infected mice (Fig. 2). All 12 of the engorged nymphs tested by PCR were positive (Table 1). Eighty-six adults were developed from the remaining nymphs. Thirty adult ticks (15 females and 15 males) were selected and analyzed for infection 4 weeks after molting. All 15 (100 %) females and 14 of the 15 (93.3 %) males were positive (*P >* 0.05).

### Transmission of *B. microti* to mice by infected nymphs

BALB/c and NOD/SCID mice were infested with infected nymphs by applying 20 nymphs to each mouse as described above. *B. microti* was detected by PCR in 2 (16.7 %) blood samples of 12 BALB/c mice and in all samples of the 6 NOD/SCID mice (*P* < 0.01, shown in Table 2). Parasitemia was earlier in NOD/SCID mice (7 to 11 days after infestation) than in BALB/c mice (13 to 17 days). However, the parasite was not found in any of the blood smears.

**Table 2:** Infection of mice with *B. microti* by infected nymphs

Mice	No. of mice applied	No. (%) of infected mice*	Prepatent period (days after infestation)
BALB/c	12	2 (16.7)	13–17
NOD/SCID	6	6 (100)	7–11

*P < 0.01

### Transstadial persistence and transovarial transmission

Infective nymphs were fed on BALB/c or NOD/SCID mice free of *B. microti*, and 337 were engorged and harvested (Fig. 1). Five of the 40 engorged nymphs detected were still *B. microti* positive (positive rate: 12.5 %, shown in Table 3). Eighty of the adult ticks that developed from these nymphs were subsequently analyzed for infection four weeks after molting, and none of them were positive. These results indicated that *B. microti* infection could not persist in the *R. haemaphysaloides* tick beyond one instar and that infection of the tick must occur at the instar preceding the transmitting stage.

**Table 3:** Transstadial persistence of *B. microti* in *R. haemaphysaloides* ticks

Stage of infected ticks	No. of blood-feeding ticks analyzed	Positive blood-feeding ticks, *n* (%)	No. of ticks detected 4 week post molting/oviposition	Positive ticks post molting/oviposition, *n* (%)
Nymph	40	5 (12.5)	80	0 (0)
Female adult	10	1 (10.0)	17	2 (11.8)

Infective adult ticks were fed on a rabbit, and 27 engorged female ticks were collected (Fig. 2). Only 10 % (1/10) of the engorged females detected without oviposition were *B. microti* positive (Table 3). Two (11.8 %) of the 17 females were positive after oviposition. All the eggs and larvae were negative.

## Discussion

Dozens of human infections of *B. microti* have been reported recently, especially in southern China [6, 9]. However, the transmission vectors of this parasite in these areas are not well understood. Moreover, *Rhipicephalus haemaphysaloides* ticks are very common and widespread in southern China [17]. Thus, investigating the transmission capability of *R. haemaphysaloides* for *B. microti* is needed.

As far as we are aware, this study is the first to investigate the transmission of *B. microti* using laboratory experiments in ticks of the genus *Rhipicephalus*. The present study confirmed the experimental transstadial transmission of *R. haemaphysaloides* for *B. microti* in two transstadial routes, i.e. larva to nymph (43.8 %) and nymph to adult (96.7 %). The transmission efficiency of *R. haemaphysaloides* from infected animals is comparable to that of *Ixodes scapularis* and *I. ricinus*, which are the primary vectors of *B. microti* in the United States and Europe [24, 25]. The results also showed that the transmission efficiency from nymph to adult was higher than that from larva to nymph (Table 1). This could be explained by nymphs ingesting much more infected blood than larvae do during feeding [26].

Consistent with other studies [24, 25], this study shows that the parasite of *B. microti* does not persist beyond more than one molt (Table 3) and that transovarial transmission does not occur. Interestingly, we also found that positive rate of tick infection declined dramatically after blood feeding (from 43.8 to 12.5 % for infected nymphs and from 100 to 10.0 % for female adults, as shown in Tables 1 and 3). The results suggest that blood-feeding induces immune response to pathogen in ticks such as other blood-sucking vectors [27, 28]. However, more investigations should be performed to understand the mechanisms underlying this phenomenon and to investigate the vector-parasite interaction in ticks.

The severity of babesiosis depends primarily on the immune status of the hosts [1]. Severe *B. microti* illness is usually seen among immunocompromised patients. Similarly, the present study showed that *B. microti* infection was much easier to establish from infective ticks in immunodeficient NOD/SCID mice than in immunocompetent BABL/C mice.

There are several limitations in our study. First, we did not witness any parasites by blood smear evaluation because the monitoring of the parasite was stopped after the first positive PCR result. Furthermore, no tick salivary glands were tested for infection in our study. These weak points reduced the confidence that our study proved the ability of *R. haemaphysaloides* as a natural vector, as nested PCR is prone to contamination. Secondly, the transmission competence of *R. haemaphysaloides* might be challenged in natural environments due to the brief duration of patent infections in wild animals as opposed to the prolonged parasitaemias produced by repeated syringe passage into inbred laboratory mice [26]. Thirdly, the transmission of *B. microti* from ticks to natural hosts of this parasite was not investigated, and their susceptibility may be quite different from laboratory animals [29]. Finally, transovarial transmission is probably more likely to occur when engorging female ticks are exposed to infection, but this cannot occur in nature because *R. haemaphysaloides* adult females do not engorge on the small rodent reservoir hosts of *B. microti*. To further understand the role of *R. haemaphysaloides* in transmission of *B. microti* in nature, further investigations are needed on the infection of questing ticks and on the transmission to *R. haemaphysaloides* ticks by chronic infections.

## Conclusion

In conclusion, our demonstration of *R. haemaphysaloides* transmission to mice suggests that this tick is a potential vector of human babesiosis in areas where it occurs and may be a matter of public health concern.

## References

[1] Vannier E, Krause PJ (2012). Human babesiosis. N Engl J Med.

[2] Hong SH, Anu D, Jeong YI, Abmed D, Cho SH, Lee WJ, Lee SE (2014). Molecular detection and seroprevalence of *Babesia microti* among stock farmers in Khutul City, Selenge Province, Mongolia. Korean J Parasitol.

[3] Zhou X, Li SG, Chen SB, Wang JZ, Xu B, Zhou HJ, Ge HX, Chen JH, Hu W (2013). Co-infections with *Babesia microti* and *Plasmodium* parasites along the China-Myanmar border. Infect Dis Poverty.

[4] Zhou X, Li S, Wang J, Huang J, Zhou H, Chen J, Zhou XN (2014). Emergence of human babesiosis along the border of China with Myanmar: Detection by PCR and confirmation by sequencing. Emerg Microbes Infect.

[5] Zhou X, Xia S, Huang J, Tambo E, Ge HX, Zhou XN (2014). Human babesiosis, an emerging tick-borne disease in the People’s Republic of China. Parasit Vectors.

[6] Yan Q, Heng P, Huaimin Z, Jizhou Y (2015). Nest-PCR identification of one human infected of *Babesia microti* in Guangxi and investigation on his colleagues (Article in Chinese). Int J Med Parasitol Dis.

[7] Jiang J, Zheng Y, Jiang R, Li H, Huo Q, Jiang B, Sun Y, Jia N, Wang Y, Ma L, Liu H, Chu Y, Ni X, Liu K, Song Y, Yao N, Wang H, Sun T, Cao W. Epidemiological, clinical, and laboratory characteristics of 48 cases of “*Babesia venatorum*” infection in China: a descriptive study. Lancet Infect Dis. 2014. 10.1016/S1473-3099(14)71046-1 25539588

[8] Sun Y, Li SG, Jiang JF, Wang X, Zhang Y, Wang H, Cao WC (2014). *Babesia venatorum* infection in child, China. Emerg Infect Dis.

[9] Vannier E, Krause PJ (2015). Babesiosis in China, an emerging threat. Lancet Infect Dis.

[10] Yabsley MJ, Shock BC (2013). Natural history of zoonotic *Babesia*: role of wildlife reservoirs. Int J Parasitol Parasites Wildl.

[11] Bown KJ, Lambin X, Telford GR, Ogden NH, Telfer S, Woldehiwet Z, Birtles RJ (2008). Relative importance of *Ixodes ricinus* and *Ixodes trianguliceps* as vectors for *Anaplasma phagocytophilum* and *Babesia microti* in field vole (*Microtus agrestis*) populations. Appl Environ Microbiol.

[12] Zamoto-Niikura A, Tsuji M, Qiang W, Nakao M, Hirata H, Ishihara C (2012). Detection of two zoonotic *Babesia microti* lineages, the Hobetsu and U.S. lineages, in two sympatric tick species, *Ixodes ovatus* and *Ixodes persulcatus*, respectively, in Japan. Appl Environ Microb.

[13] Sun Y, Liu G, Yang L, Xu R, Cao W (2008). *Babesia microti*-like rodent parasites isolated from *Ixodes persulcatus* (Acari: Ixodidae) in Heilongjiang Province, China. Vet Parasitol.

[14] Saito-Ito A, Yano Y, Dantrakool A, Hashimoto T, Takada N (2004). Survey of rodents and ticks in human babesiosis emergence area in Japan: first detection of *Babesia microti*-like parasites in *Ixodes ovatus*. J Clin Microbiol.

[15] Rar VA, Epikhina TI, Livanova NN, Panov VV (2011). Genetic diversity of *Babesia* in *Ixodes persulcatus* and small mammals from North Ural and West Siberia, Russia. Parasitology.

[16] Fan D, Li M, Xu H, Hu M, Zhang J, Sun Y (2012). The situation of mice and ticks infected by *Babesia microti* (Article in Chinese). Chin J Hyg Insect Equip.

[17] Chen Z, Yang X, Bu F, Yang X, Yang X, Liu J (2010). Ticks (Acari: Ixodoidea: Argasidae, Ixodidae) of China. Exp Appl Acarol.

[18] Wojcik-Fatla A, Bartosik K, Buczek A, Dutkiewicz J (2012). *Babesia microti* in adult *Dermacentor reticulatus* ticks from eastern Poland. Vector Borne Zoonotic Dis.

[19] Yu X, Gong H, Zhou Y, Zhang H, Cao J, Zhou J (2015). Differential sialotranscriptomes of unfed and fed *Rhipicephalus haemaphysaloides*, with particular regard to differentially expressed genes of cysteine proteases. Parasit Vectors.

[20] Yin H, Lu W, Luo J (1997). Babesiosis in China. Trop Anim Health Prod.

[21] Bhat HR, Naik SV, Ilkal MA, Banerjee K (1978). Transmission of Kyasanur Forest disease virus by *Rhipicephalus haemaphysaloides* ticks. Acta Virol.

[22] Ruebush MJ, Hanson WL (1979). Susceptibility of five strains of mice to *Babesia microti* of human origin. J Parasitol.

[23] Zamoto A, Tsuji M, Wei Q, Cho SH, Shin EH, Kim TS, Leonova GN, Hagiwara K, Asakawa M, Kariwa H, Takashima I, Ishihara C (2004). Epizootiologic survey for *Babesia microti* among small wild mammals in northeastern Eurasia and a geographic diversity in the BETA-Tubulin gene sequences. J Vet Med Sci.

[24] Oliveira MR, Kreier JP (1979). Transmission of *Babesia microti* using various species of ticks as vectors. J Parasitol.

[25] Gray J, von Stedingk LV, Gurtelschmid M, Granstrom M (2002). Transmission studies of *Babesia microti* in *Ixodes ricinus* ticks and gerbils. J Clin Microbiol.

[26] Randolph SE (1995). Quantifying parameters in the transmission of *Babesia microti* by the tick *Ixodes trianguliceps* amongst voles (*Clethrionomys glareolus*). Parasitology.

[27] Pakpour N, Akman-Anderson L, Vodovotz Y, Luckhart S (2013). The effects of ingested mammalian blood factors on vector arthropod immunity and physiology. Microbes Infect.

[28] Kopacek P, Hajdusek O, Buresova V, Daffre S (2010). Tick innate immunity. Adv Exp Med Biol.

[29] Piesman J, Spielman A (1982). *Babesia microti*: infectivity of parasites from ticks for hamsters and white-footed mice. Exp Parasitol.

